# Different exercise training modalities similarly improve heart rate variability in sedentary middle-aged adults: the FIT-AGEING randomized controlled trial

**DOI:** 10.1007/s00421-022-04957-9

**Published:** 2022-05-10

**Authors:** Ginés Navarro-Lomas, Manuel Dote-Montero, Juan M. A. Alcantara, Abel Plaza-Florido, Manuel J. Castillo, Francisco J. Amaro-Gahete

**Affiliations:** 1 EFFECTS-262 Research Group, Department of Physiology, Faculty of Medicine, University of Granada, Avda. de la Investigación 11, 18016 Granada, Spain; 2 1 PROmoting FITness and Health Through Physical Activity Research Group (PROFITH), Sport and Health University Research Institute (iMUDS), Department of Physical and Sports Education, Faculty of Sport Sciences, University of Granada, Camino de Alfacar s/n, 18071 Granada, Spain

**Keywords:** Autonomic nervous system, Vagal activity, physical activity, Training, Cardiometabolic health

## Abstract

**Purpose:**

This study aimed to investigate the influence of different exercise training modalities on heart rate variability (HRV) in sedentary middle-aged adults; and to study whether changes in health-related outcomes (i.e., body composition and cardiometabolic risk) are associated with those hypothetical HRV changes in sedentary middle-aged adults.

**Methods:**

A total of 66 middle-aged adults (53.6 ± 4.4 years old; 50% women) were enrolled in the FIT-AGEING study. We conducted a 12-week randomized controlled trial. The participants were randomly assigned to 4 groups: (a) a control group (no exercise); (b) a physical activity recommendation from the World Health Organization group (PAR); (c) a high-intensity interval training group (HIIT); and (d) a high-intensity interval training group adding whole-body electromyostimulation (HIIT + EMS).

**Results:**

All exercise training modalities induced changes in HRV parameters (all *P* ≤ 0.001) without statistical differences between them (all *P* > 0.05). We found associations between changes in body composition and cardiometabolic risk and exercise-related changes in HRV.

**Conclusion:**

Our results suggest that different exercise interventions (i.e., PAR, HIIT and HIIT + EMS) induced an enhancement of HRV in sedentary middle-aged adults. Our findings support the notion that exercise-related changes in HRV are associated with changes in body composition and cardiometabolic risk after the intervention program

**Clinical trial registry:**

NCT03334357 (ClinicalTrials.gov). November 7, 2017 retrospectively registered.

**Supplementary Information:**

The online version contains supplementary material available at 10.1007/s00421-022-04957-9.

## Introduction

Heart rate variability (HRV) describes the time differences between successive R–R intervals (Task force of the European Society of Cardiology and the North American Society for Pacing and Electrophysiology 1996; Navarro-Lomas et al. 2020). HRV is commonly used as a non-invasive method to describe the influence of the autonomic nervous system on heart function (Almeida-Santos et al. 2016; Navarro-Lomas et al. 2020; Wong and Figueroa 2021). Concretely, HRV is a psycho-physiological phenomenon with broad implications (Dantas et al. 2018), including physiological, neuro-psychological, pathological, environmental and lifestyle factors (Zulfiqar et al. 2010; Ernst 2017; Navarro-Lomas et al. 2020). Interestingly, previous studies have reported that decreased vagal-related HRV parameters [e.g., RMSSD (square root of the mean squared differences between successive RR intervals)] during resting conditions are associated with a higher incidence of several chronic cardiometabolic diseases (e.g., obesity or type 2 diabetes), while the increase of these HRV parameters is related to a healthy status and reduced levels of stress (Tsuji et al. 1996; Navarro-Lomas et al. 2020). Hence, a reduced HRV during resting conditions is associated with an increased risk of a first cardiovascular event in populations without known cardiovascular disease (Hillebrand et al. 2013). Furthermore, a decrease in vagal-related HRV parameters has been related to ageing processes, especially from 40 to 60 years (Plaza-Florido et al. 2020).

Physical exercise is currently considered a promising strategy to improve HRV (i.e., increase vagal-related HRV parameters during resting) (Felber Dietrich et al. 2008; Wong and Figueroa 2021). In addition, low levels of physical activity have been linked to a higher risk of cardiovascular disease, obesity and co-morbidities, and all-cause mortality (Alansare et al. 2018; Fiuza-Luces et al. 2018; Amaro-Gahete et al. 2019a). Nonetheless, the majority of people in developed societies do not meet physical activity recommendations from the World Health Organization arguing the lack of time as the main reason (Gómez-López et al. 2010; Choi et al. 2017). In this context, less time-consuming training methodologies have recently emerged, such as high-intensity interval training (HIIT) or whole-body electromyostimulation training (EMS) (Amaro-Gahete et al. 2019a) obtaining from them and its combination promising results on body composition, cardiometabolic risk factors (i.e., metabolic syndrome markers) and other health-related outcomes in sedentary, but healthy, middle-aged adults (Amaro-Gahete et al. 2019a; b, c).

A long-term aerobic exercise intervention has been proposed as an effective tool to increase vagal-related HRV parameters during resting conditions in adults (Tulppo et al. 2003; Albinet et al. 2010). Furthermore, an HIIT program seems to induce better adaptations in HRV compared with those obtained in response to a moderate-intensity continuous training in physically inactive adults aged between 20 and 40 years (Alansare et al. 2018). Interestingly, a 6-week EMS intervention did not promote additional benefits on HRV in addition to those observed from a similar exercise training alone in obese patients aged between 18 and 50 years after bariatric surgery (Ricci et al. 2020). Nevertheless, EMS effects over HRV parameters have not been previously studied in sedentary middle-aged adults (45–65 years old). Moreover, to the best of our knowledge, no previous studies compared the effects of 3 different exercise training modalities on HRV in sedentary middle-aged adults. Therefore, the present study aimed (i) to investigate the influence of different exercise training modalities [i.e., (a) concurrent training (aerobic + resistance training) based on physical activity recommendation from the World Health Organization (PAR); (b) HIIT; and (c) HIIT + EMS] on HRV in sedentary middle-aged adults; and (ii) to study whether changes in health-related outcomes (i.e., body composition and cardiometabolic risk) are associated with those hypothetical HRV changes in sedentary middle-aged adults. We hypothesized (i) that all exercise interventions will induce significant improvements in HRV, especially in HIIT and HIIT + EMS groups; and (ii) that exercise-related changes in HRV will be related to changes in body composition and cardiometabolic risk in sedentary, middle-aged adults.

## Methods

### Participants

A total of 66 middle-aged adults (45–65 years old; 50% women) were enrolled in the FIT-AGEING randomized controlled trial (clinicaltrial.gov: ID: CT03334357) (Amaro-Gahete et al. 2018). Participants were sedentary (i.e., < 20 min of physical activity on < 3 days/week during the last 3 months) and healthy individuals from the province of Granada (Spain). The inclusion criteria were (i) to have a stable body weight over the previous 3 months (i.e., changes < 5 kg) and (ii) to have no chronic metabolic disease, cancer, or any health problem that could be aggravated by physical activity. Moreover, a medical examination was done before the intervention program.

### Ethics statement

The study procedures were approved by the Human Research Ethics Committee of the “Junta de Andalucía” [0838- N-2017] and followed the principles of the last revised Declaration of Helsinki (7th revision of October 2013). All participants provided written informed consent.

### Study design

We conducted a 12-week randomized controlled trial with a parallel-group design following the CONSORT (Consolidated Standards of Reporting Trials) guidelines (see Supplementary Material, Table S1). After completing the baseline (i.e., pre-exercise intervention) measurements (see Amaro-Gahete et al. (Amaro-Gahete et al. 2018) for detailed information), the participants were randomized into four different groups using a computer-generated simple randomization software: (a) control group (no exercise), (b) PAR group, (c) HIIT group, and (d) HIIT + EMS group. All participants were instructed to maintain their usual physical activity levels, lifestyle habits and not to be engaged in other structured exercise interventions outside of the assigned program. No dietary prescriptions or instructions to the participants in the control and exercise groups were provided. The participants were asked to maintain their dietary habits during the intervention period.

### Training modalities

We have previously reported a detailed description of each training modality in a methodological manuscript (Amaro-Gahete et al. 2018). In brief, each session started with a dynamic standardized warm-up and finished with an active global stretching cooling-down protocol in all intervention groups. To be included in the per protocol analysis, an attendance of at least 90% of sessions was required.

PAR group completed three concurrent training (i.e., combination of endurance and resistance training) sessions per week for 12 weeks with at least 48 h of recovery between sessions. Endurance training consisted of 150 min/week (organized in 10 min bouts) at 60–65% of their heart rate reserve. Resistance training was performed at 40–50% of one-repetition maximum. Different ergometers (i.e., treadmill, cycle-ergometer, and elliptical ergometer) were selected to conduct the endurance training, and weight-bearing and guided pneumatic machines were selected to conduct the resistance training (i.e., squat, bench press, dead lift, and lateral pull-down). Also, compensatory exercises (e.g., flexibility or core stability) were realized to minimize the risk of injuries.

HIIT group performed an intervention program characterized by short and intermittent efforts of high intensity, interspersed with rest periods at passive or low-intensity exercises. The participants performed a total of 2 sessions/week for 12 weeks following 2 different complementary protocols: (i) high-intensity interval training with long intervals (type A session), and (ii) high-intensity interval training with short intervals (type B session). The training volume was 40–65 min/week at > 95% of the maximum oxygen uptake (VO_2_max) in type A sessions, and > 120% of the VO_2_max in type B sessions. Type A sessions had a maximal duration of 24 min/session, where participants completed 6–10 sets of 4 or 5 min (2- or 3-min work/2-min rest). Type B sessions had a maximal duration of 37 min/session, where participants performed 2 or 3 sets (8–12.5 min of duration) of 16 exercises (15–30 s work/15–30 s rest) with an active rest of 5 min at 60% between sets. The exercise program for type A sessions was based on walking on a treadmill with a personalized slope, and type B sessions included a circuit workout with 8 weight-bearing exercises (i.e., squat, dead lift, high knees up, high heels up, push up, horizontal row, lateral plank, and frontal plank).

HIIT + EMS group completed a training program that followed the same structure that HIIT in terms of the type of exercise, training intensity, training frequency and training volume including EMS to check whether this additional stimulus induces an extra effect on HRV parameters. Bipolar, symmetrical, and rectangular electric pulse was applied with: (a) a frequency of 15‐20 Hz in type A sessions, and 35‐75 Hz in type B sessions; (b) an intensity of 100 milliamps in type A sessions, and 80 milliamps in type B sessions; (c) an impulse breadth of 200‐400 µs (thigh zone = 400 μs, glute zone = 350 μs, abdominal zone = 300 μs, dorsal zone = 250 μs, cervical zone = 200 μs, chest zone = 200 μs, and arm zone = 200 μs); and (d) a duty cycle (ratio of on‐time to the total cycle time: % duty cycle = 100/ [total time/ on‐time]) of 99% in type A sessions, and 50%‐63% in type B sessions. A whole‐body electromyostimulation device manufactured by Wiemspro® (Malaga, Spain) was used.

### HRV

Participants came to our laboratory, by motorized vehicle and avoiding any physical activity since they woke up, between 8.00 and 10.00 a.m. following specific study pre-conditions: (i) fasting conditions; (ii) not altered sleep pattern the night before; (iii) to be abstained from alcohol intake and drugs or stimulant consumption, including coffee and other stimulants 24 h before; and (iv) to avoid moderate-intensity physical activity (24 h) and vigorous-intensity physical activity (48 h) before the test. The environmental conditions were standardized (room temperature = 22–23 °C).

The assessment of the R–R signal was carried out with the participant lying on a stretcher in the supine position. To obtain a repeatable HRV measure, R–R signal recording lasted 15 min (after 10 min of acclimation) (Schroeder et al. 2004). We used the Polar RS800CX (Polar Electro, Kempele, Finland) to record the R–R signal. In previous studies, polar RS800CX has been showed as a reliable (Intraclass Correlation Coefficients for test–retest reliability ranges from 0.29 to 0.61) (Vasconcellos et al. 2015) and valid (Pearson correlation coefficient < 0.97 compared with high-quality electrocardiogram) (Gamelin et al. 2008) tool for HRV assessment. Participants were instructed not to talk or to move, and to relax as much as possible but being awake. R–R recordings were downloaded by the Polar Pro Trainer 5^®^ software (Polar Electro, Finland) and were analyzed with the Kubios HRV Standard^®^, free version 2.2 software (University of Eastern Finland, Kuopio, Finland) (Tarvainen et al. 2014), following the methodology described in previous studies and applying the medium filter provided by the Kubios HRV Standard^®^ (Shaffer and Ginsberg 2017; Plaza-Florido et al. 2021). The smoothness prior approach with a Lambda value of 500, and a cubic interpolation at the default rate of 4 Hz, was used to remove not valid low-frequency baseline trend components. Considering the findings obtained in a previous study that analyzed the quantification of the inter-researcher (Intraclass Correlation Coefficient ranges from 0.850 to 0.987, Coefficient of Variation lower than 21.1%) and intra-researcher (Intraclass Correlation Coefficient ranges from 0.951 to 0.995, Coefficient of Variation lower than 13.7%) differences in HRV of the present cohort, HRV analyses were conducted by the same trained researcher to obtain reproducible and valid data (Plaza-Florido et al. 2020).

HRV parameters in Time-Domain [i.e., SDNN (standard deviation of RR intervals) and RMSSD] and non-linear analyses (i.e., Poincare plot) were derived by the HRV Kubios software following standard procedures (Task force of the European Society of Cardiology and the North American Society for Pacing and Electrophysiology 1996). SDNN is an indicator of global autonomic modulation (Shaffer and Ginsberg 2017), while RMSSD is a contrasted marker of vagal modulation (Shaffer and Ginsberg 2017). Poincare plot analysis is considered an indicator of heart rate complexity (Tayel and AlSaba 2015). SD1 (standard deviation of Poincare plot orthogonal to the line-of-identity) and SD2 (standard deviation of Poincare plot along the line-of-identity) were obtained from Poincare plot analysis. SD1 has been positioned as vagal activity indicator, while SD2 is inversely related to sympathetic activity (Naranjo-Orellana et al. 2015; Navarro-Lomas et al. 2020). Stress Score (SS), calculated as 1000*1/SD2, and sympathetic/parasympathetic ratio (S/PS ratio), computed as SS/SD1, were also determined (Naranjo-Orellana et al. 2015; Navarro-Lomas et al. 2020). SS has been associated with sympathetic activity (Naranjo-Orellana et al. 2015; Navarro-Lomas et al. 2020), whereas S/PS ratio is considered an indicator of autonomic balance (Naranjo-Orellana et al. 2015; Navarro-Lomas et al. 2020). Also, we included in supplementary material (Fig. S1) other HRV parameters, in Time-Domain [i.e., PNN50 (percentage of successive intervals that differ more than 50 ms; a vagal-related HRV parameter)] and Frequency-Domain [i.e., high frequency (0.15–0.40 Hz; a measure of vagal activity (Shaffer and Ginsberg 2017)), low frequency (0.04–0.15 Hz; a measure of baroreflex activity (Shaffer and Ginsberg 2017)) and low/high frequency ratio (traditionally used to estimate sympathovagal balance, although its interpretation depends on specific measurement conditions (Shaffer and Ginsberg 2017))].

To remove the HRV dependence on heart rate, we calculated corrected HRV parameters (Plaza-Florido et al. 2021) (see Supplementary Material, Fig. S2). For that purpose, three assumptions were considered: (i) if HRV parameters were negatively correlated with heart rate, the correction procedure consisted in calculating ratios between HRV parameters and different powers of the mean R–R interval; (ii) if HRV parameters were positively correlated with heart rate, the correction procedure was performed by multiplying HRV parameters by the adequate powers of mean R–R interval; and (iii) pre- and post-values of the different HRV parameters were normalized with the same powers of mean R–R intervals to calculate post–pre differences: corrected SDNN = SDNN/MeanRR^1.6^, Corrected RMSSD = RMSSD/MeanRR^2.7^, Corrected SS = SS*Mean RR^1.1^ and Corrected S/PS ratio = S/PS ratio*MeanRR.

### Anthropometry and body composition

We measured height and weight using an electronic scale (model 799, Electronic Column Scale, Hamburg, Germany). Waist circumference was assessed twice at the midpoint between the iliac crest and the bottom of the rib cage at the end of a normal expiration. Fat mass, lean mass and visceral adipose tissue volume (VAT) were assessed by dual-energy X-ray absorptiometry (Discovery Wi, Hologic, Inc., Bedford, MA, USA). We also calculated 3 height-normalized body composition indices: body mass index (BMI), as body weight/height^2^; lean mass index (LMI), as lean mass/height^2^; and fat mass index (FMI), as fat mass/height^2^.

### Blood pressure

Blood pressure was measured in the right arm with participants resting in a sitting position using an HEM 705 CP automatic monitor (Omron Healthcare Co., Kyoto, Japan) following the guidelines of the European Heart Society (Carey et al. 2018). Readings were taken twice and the mean was subsequently recorded. Mean blood pressure was defined as systolic blood pressure minus 1/3 of the diastolic blood pressure (Carey et al. 2018).

### Blood parameters

All samples were collected between 8:30 and 10 am after an overnight fast (12 h) and a 10-min rest in the supine position. We obtained blood samples from the antecubital vein. Samples were collected in prechilled ethylene diamine tetra-acetic acid-containing tubes (Vacutainer SST, Becton Dickinson, Plymouth, UK), and immediately centrifuged (i.e., 15 min at 3000 rpm), aliquoted and stored at − 80 °C until analysis. Glucose, high-density lipoprotein cholesterol (HDL-C) and triglycerides were assessed using a spectrophotometer (model AU5800, Beckman Coulter, Brea, CA, USA). HRV and blood parameters were measured on different days.

### Cardiometabolic risk

A sex-specific cardiometabolic risk score was calculated based on the clinical variables suggested by the International Diabetes Federation and the Adult Treatment Panel III for defining metabolic syndrome (i.e., waist circumference, mean blood pressure, plasma glucose, HDL-C and triglycerides) (Carracher et al. 2017). Each variable was represented in a standardized mode as (value-mean)/standard deviation. The standardized HDL-C values were multiplied by − 1 to indicate greater risk with increasing values. The cardiometabolic risk score was calculated as the sum of these 5 standardized values divided by 5, obtaining a mean of 0 and a standard deviation of 1 by definition. Therefore, lower values represent a better cardiometabolic risk profile (Carracher et al. 2017).

### Statistical analysis

To verify the distribution of all variables we used the Shapiro–Wilk test, Q–Q plots and visual check of histograms. The descriptive parameters are reported as mean (standard deviation), excepting SDNN, RMSSD, SS and S/PS ratio, that were presented as median and interquartile range since they do not exhibit a normal distribution. Thereafter, these HRV parameters were normalized using Napierian logarithm transformation. Before the intervention program, we performed an analysis of variance (ANOVA) to study baseline differences in heart rate, HRV parameters, body composition, and cardiometabolic risk between intervention groups. Analysis of covariance (ANCOVA) was employed to study the effect of the different intervention groups on HRV parameters controlling for the HRV baseline values. All group-related changes were additionally adjusted by sex and age. Bonferroni post hoc tests with adjustment for multiple comparisons were performed to determine differences between all exercise modality groups. *F* (analysis of variance), *P* (level of significance, *η*^2^ (partial eta squared) and 95% confidence interval were reported from these analyses. To examine the relationship of changes in HRV parameters with changes in body composition variables and cardiometabolic risk, we conducted a multiple linear regression analysis based on the post–pre differences, adjusting by sex and age. *β* (standardized regression coefficient), *R*^2^ (adjusted determination coefficient) and *P* (level of significance) were obtained from these linear regression analyses. *P* values of less than 0.05 were accepted to indicate statistical significance. All analyses were performed using the Statistical Package for Social Sciences (SPSS, v. 24.0, IBM SPSS Statistics, IBM Corporation). The figures were created using GraphPad Prism 7 (GraphPad Software, San Diego, CA, USA).

## Results

**Fig. 1:**
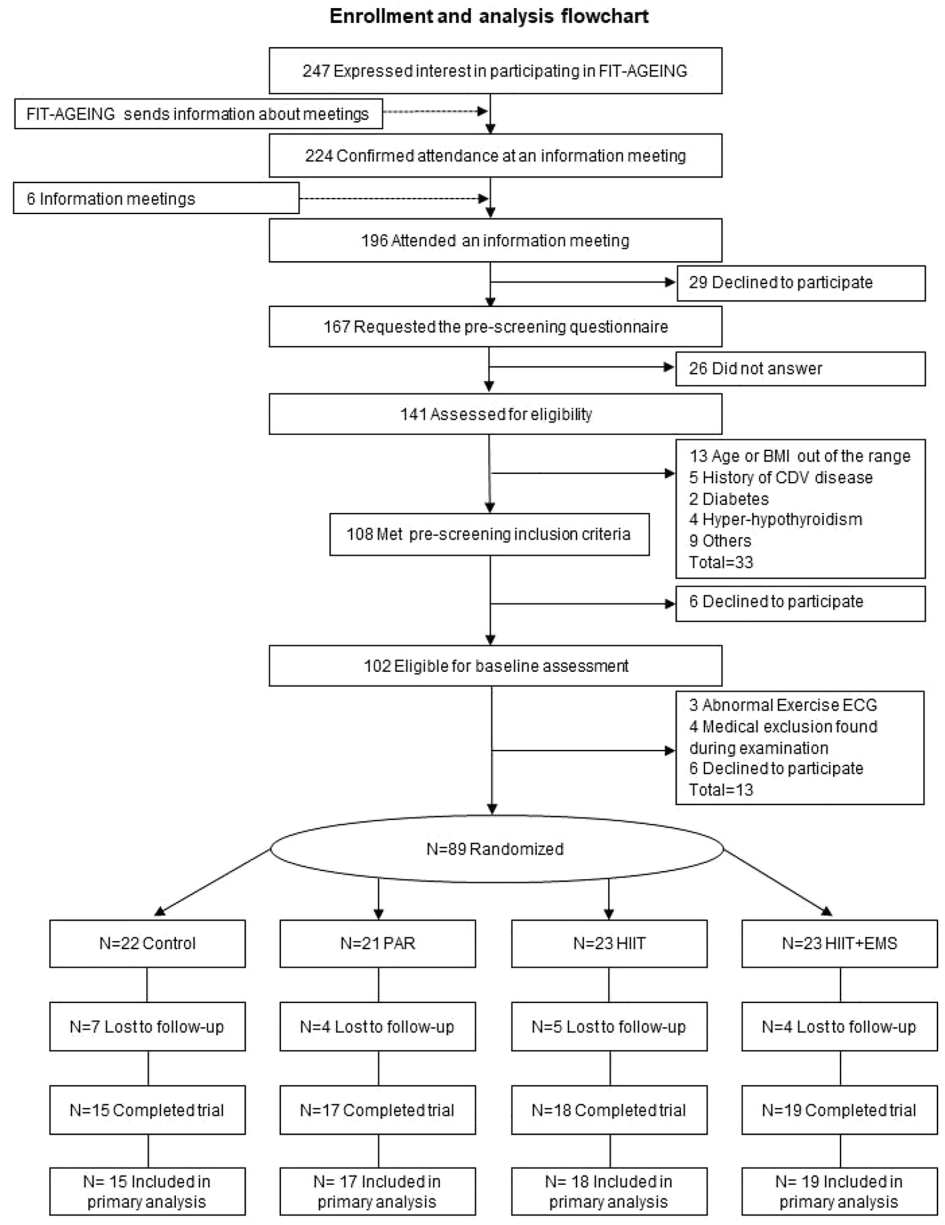
Enrollment and analysis flowchart. Abbreviations: *BMI* body mass index; *CDV *cardiovascular disease; *ECG* electrocardiogram; *PAR* physical activity recommendation from the World Health Organization group; *HIIT* high-intensity interval training group; HIIT + EMS—HIIT plus whole-body electromyostimulation group

Figure 1 shows the flowchart of the FIT-AGEING study. A total of 66 participants (*n* = 13 in control, *n* = 16, in PAR, *n* = 18 in HIIT and *n* = 19 in HIIT + EMS) were included in the per protocol analyses.

The baseline characteristics of all participants are described in Table 1. No differences were observed in the baseline values among groups.

**Table 1:** Descriptive values by intervention group in baseline

	All (*n* = 69)	Control (*n* = 15)	PAR (*n* = 17)	HIIT (*n* = 18)	HIIT + EMS (*n* = 19)	*P* value
Age (years)	53.4 (5.0)	51.7 (4.1)	53.9 (4.5)	53.1 (5.6)	53.5 (5.2)	0.326
Sex (%)						
Men	33 (47.8)	6 (40)	8 (47)	9 (50)	10 (53)	
Women	36 (52.2)	9 (60)	9 (53)	9 (50)	9 (47)	
HR and HRV						
Heart rate (bpm)	63.3 (8.3)	63.8 (6.7)	59.9 (7.6)	64.5 (7.6)	64.5 (10.3)	0.317
SDNN (ms)	27.3 (18.6)	31.0 (24.5)	23.4 (16.6)	29.8 (18.5)	24.4 (16.8)	0.291
RMSSD (ms)	26.4 (20.7)	33.4 (18.1)	23.8 (20.5)	29.1 (17.0)	24.6 (36.3)	0.959
Stress score (ms)	30.6 (20.7)	26.5 (20.3)	35.2 (18.6)	27.3 (15.7)	35.7 (32.2)	0.174
Sympathetic/parasympathetic ratio	1.53 (2.62)	1.12 (2.13)	2.13 (2.35)	1.32 (2.45)	1.83 (6.68)	0.128
Anthropometry and body composition						
Weight (kg)	76.3 (8.7)	74.1 (13.8)	72.6 (11.3)	78.2 (17.7)	80.2 (15.9)	0.412
Waist circumference (cm)	95.2 (12.0)	92.8 (10.5)	90.4 (11.0)	97.5 (10.9)	99.3 (13.7)	0.101
Body mass index (kg/m^2^)	26.84 (3.83)	26.73 (3.89)	25.41 (2.86)	26.43 (3.15)	28.60 (14.64)	0.083
Lean mass index (kg/m^2^)	1.10 (0.10)	15.85 (3.11)	15.16 (2.48)	14.90 (2.88)	15.80 (2.88)	0.694
Fat mass index (kg/m^2^)	15.42 (2.81)	10.09 (2.68)	9.60 (2.73)	10.79 (2.67)	11.96 (3.78)	0.115
VAT (cm^3^)	849.7 (427.2)	744.9 (288.8)	714.9 (283.9)	879.6 (488.9)	1024.6 (517.3)	0.116
Cardiometabolic risk score						
Mean blood pressure (mmHg)	104.2 (12.4)	105.4 (13.4)	105.3 (13.8)	103.6 (13.8)	102.9 (12.1)	0.927
Plasma glucose (mg/dL)	93.9 (11.4)	94.9 (10.7)	93.4 (11.6)	90.1 (5.6)	96.9 (14.8)	0.332
Plasma insulin (UI/mL)	8.06 (5.75)	7.03 (5.28)	7.52 (3.97)	7.09 (4.51)	10.21 (7.88)	0.289
HDL-C (mg/dL)	58.7 (12.3)	61.2 (12.1)	55.2 (12.0)	57.8 (10.8)	60.6 (14.0)	0.477
Triglycerides (mg/dL)	130.8 (65.2)	131.2 (72.3)	130.9 (70.0)	134.1 (61.5)	127.6 (63.3)	0.994
Cardiometabolic Risk Score	0.002 (0.58)	− 0.040 (0.68)	− 0.008 (0.52)	− 0.030 (0.47)	0.073 (0.68)	0.940

Data are shown as means (standard deviation). Median (IQR: interquartile range) are presented for HRV parameters because these variables presented non-normal distribution. Abbreviations: *bpm* beats per minute, *dL* deciliters, cm; centimeters, *cm*^*2*^ centimeters square, *cm*^*3*^ cubic centimeters, *HIIT* high-intensity interval training group; *HIIT + EMS* HIIT plus whole-body electromyostimulation group, *HR* heart rate, *HRV* heart rate variability, *mg* milligrams, *mmHG* millimeters of mercury, *ms* milliseconds, *P value* one-way ANOVA (to detect between-group differences at baseline), *PAR* physical activity recommendation from the World Health Organization group, *pg* picograms, *RMSSD* square root of the mean squared differences between successive RR intervals, *SDNN* standard deviation of RR intervals, *VAT* visceral adipose tissue

**Fig. 2:**
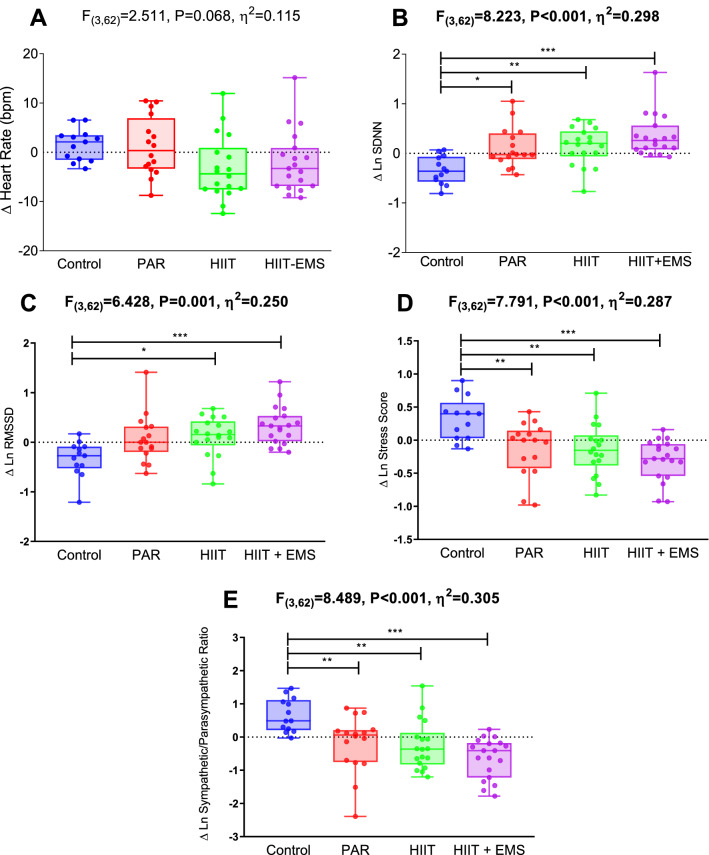
Changes in heart rate and heart rate variability parameters after the intervention study in the four groups. Analysis of covariance adjusting for baseline values, with post hoc Bonferroni-corrected *t* test. *F*, *p* and *η*^*2*^ of analysis of variance, *p < 0.05, **p < 0.01, ***p < 0.001. Abbreviations: ∆ Differences between post and pretest, *bpm* beats per minute, *HIIT* high-intensity interval training group; *HIIT + EMS* HIIT plus whole-body electromyostimulation group, *Ln* Napierian logarithm, *PAR* physical activity recommendation from the World Health Organization group, *RMSSD* square root of the mean squared differences between successive RR intervals, *SDNN* standard deviation of RR intervals

Figure 2 shows changes in HRV parameters after the intervention study in the four groups. We found a significant difference in SDNN (Fig. 2B; *F* = 8.223, *P* < 0.001, *η*^2^ = 0.298), RMSSD (Fig. 2C; *F* = 6.428, *P* = 0.001, *η*^2^ = 0.250), SS (Fig. 2D; *F* = 7.791, *P* < 0.001, *η*^2^ = 0.287) and S/PS ratio (Fig. 2E; *F* = 8.489, *P* < 0.001, *η*^2^ = 0.305) among groups, while no differences were noted in heart rate (Fig. 2A; *F* = 2.511, *P* = 0.068, *η*^2^ = 0.115). Compared with the control group, the SDNN levels (Fig. 2B) were increased in PAR [mean difference (95% confidence interval): 0.454 (0.068, 0.840), *P* = 0.013], HIIT [0.480 (0.104, 0.857), *P* = 0.006] and HIIT + EMS [0.688 (0.315, 1.060), *P* < 0.001]; while the RMSSD levels (Fig. 2C) were also improved in HIIT [0.443 (0.038, 0.847), *P* = 0.024], and HIIT + EMS [0.667 (0.266, 1.066), *P* < 0.001]. Compared with the control group, the SS levels (Fig. 2D) were reduced in PAR [− 0.457 (− 0.822, − 0.092), *P* = 0.007], HIIT [− 0.469 (− 0.826, − 0.113), *P* = 0.004], and HIIT + EMS [− 0.623 (− 0.976, − 0.271), *P* < 0.001], while the S/PS levels (Fig. 2E) were also reduced in PAR [− 0.864 (− 1.570, − 0.158), *P* = 0.009], HIIT [− 0.913 (− 1.601, − 0.225), *P* = 0.004] and HIIT + EMS [− 1.291 (− 1.971, − 0.610), *P* < 0.001]. The results persisted after including sex and age in the model. The raw changes (i.e., without Napierian logarithm transformation) in heart rate and heart rate variability parameters [i.e., SDNN, RMSSD, PNN50, high frequency, low frequency, low/high frequency ratio, SS and S/PS ratio] after the intervention study in the four groups are shown in Supplementary Material (Fig. S1). Supplementary Fig. S2 shows the raw changes in corrected HRV parameters after the intervention. Corrected SDNN (Fig. S2A; *F* = 7.626, *P* < 0.001, *η*^2^ = 0.273) and Corrected SS (Fig. S2C; *F* = 3.826, *P* = 0.014, *η*^2^ = 0.161) maintained significant changes in post–pre differences (*p* < 0.05), while a tendency of changes was observed for corrected RMSSD (Fig. S2B; *F* = 2.511, *P* = 0.067, *η*^2^ = 0.112). In contrast, Corrected S/PS ratio did not change after the intervention (Fig. S2D; *F* = 0.620, *P* = 0.605, *η*^2^ = 0.032).

Table 2 shows the multiple linear regression analysis of exercise-related changes in body composition and cardiometabolic risk with exercise-related changes in HRV parameters. A significant association (*β* ranges from − 0.414 to 0.387; *R*^2^ ranges from 0.072 to 0.193; all *P* < 0.05) was noted of changes in body composition (i.e., increments in LMI and reductions in FMI and VAT) with changes in HRV parameters (i.e., increments in SDNN and RMSSD and reductions in SS and S/PS ratio). Also, we found a significant relation (*β* ranges from − 0.380 to 0.330; *R*^2^ ranges from 0.072 to 0.131; all *P* < 0.05) between decrements in Cardiometabolic Risk Score with changes in HRV parameters (i.e., increments in SDNN and reductions in SS and S/PS ratio). No significant relationship was observed between changes in BMI and changes in SDNN (*P* = 0.961), RMSSD (*P* = 0.686), SS (*P* = 0.686) and S/PS ratio (*P* = 0.809). The results persisted after including sex and age in the model.

**Table 2:** Simple linear regression analysis between changes in heart rate variability outcomes and changes in, body composition variables and cardiometabolic risk score

	∆ heart rate (bpm)			∆ Ln SDNN (ms)			∆ Ln RMSSD (ms)			∆ Ln stress score (ms)			∆ Ln S/PS ratio		
	*β*	*R*^2^	*P*	β	*R*^2^	*P*	*β*	*R*^2^	*P*	*β*	*R*^2^	*P*	*β*	*R*^2^	*p*
∆ BMI (kg/m^2^)	− 0.025	< 0.001	0.844	− 0.006	< 0.001	0.961	0.101	< 0.001	0.419	0.051	< 0.001	0.686	− 0.030	< 0.001	0.809
∆ LMI (kg/m^2^)	− 0.182	0.017	0.153	**0.387**	**0.150**	**0.001**	**0.439**	**0.193**	** < 0.001**	**− 0.326**	**0.107**	**0.009**	**− 0.414**	**0.172**	** < 0.001**
∆ FMI (kg/m^2^)	0.157	0.009	0.220	**− 0.347**	**0.121**	**0.005**	**− 0.370**	**0.137**	**0.003**	**0.299**	**0.090**	**0.017**	**0.362**	**0.072**	**0.003**
∆ VAT volume (cm^3^)	0.142	0.004	0.269	**− 0.293**	**0.086**	**0.019**	**− 0.295**	**0.082**	**0.023**	**0.267**	**0.072**	**0.034**	**0.299**	**0.090**	**0.017**
∆ Cardiometabolic Risk Score	− 0.114	< 0.001	0.362	**− 0.330**	**0.095**	**0.007**	− 0.176	0.016	0.158	**0.380**	**0.131**	**0.002**	**0.293**	**0.072**	**0.017**

Bold values indicated *P* values of less than 0.05

∆ differences between post and pretest, *β* standardized regression coefficient, *BMI* body mass index, *bpm* beats per minute, *cm* centimeters, *FMI* fat mass index, *kg* kilograms, *LMI* lean mass index, *Ln* Napierian logarithm, *m* meters, *p* level of significance, *R*^*2*^ adjusted determination coefficient, *RMSSD* square root of the mean squared differences between successive RR intervals, *SDNN* standard deviation of RR intervals, *S/PS* sympathetic/parasympathetic ratio, *VAT* visceral adipose tissue

## Discussion

This study shows that different exercise training interventions (i.e., PAR, HIIT and HIIT + EMS) similarly improved HRV parameters (i.e., increments of SDNN and RMSSD and decrements of SS and S/PS ratio) during resting conditions in sedentary middle-aged adults. In addition, these exercise-related changes in HRV parameters were associated with exercise-related changes in body composition and cardiometabolic risk. These findings have important clinical implications since we demonstrate that a well-designed 12-week exercise intervention—independently of its modality—could be an adequate stimulus to improve cardiac autonomic modulation during resting conditions (i.e., increase of the vagal modulation), which is related to a lower risk of cardiovascular disease and mortality in sedentary, healthy middle-aged adults (Lahiri et al. 2008).

Physical exercise has been postulated as an effective strategy to improve cardiac autonomic function (Felber Dietrich et al. 2008; Wong and Figueroa 2021). We do not observe significant changes in resting heart rate after the intervention, although a tendency toward a reduction in resting heart rate (i.e., bradycardia) was observed in all training groups compared with the control group (∼4%). In this sense, physical exercise (including high-intensity training) has been related to bradycardia (Cornelissen et al. 2010), thus improving cardiac function through a lower intrinsic heart rate (Bahrainy et al. 2016). Concerning HRV parameters, a 12-week PAR training at moderate intensity induces health-related changes in different HRV parameters (i.e., increases in SDNN and RMSSD; decreases in SS; and reductions with a dependence of heart rate in S/PS ratio) in sedentary middle-aged adults. Previous studies reported similar changes in HRV parameters after a 6-month moderate-intensity training program in sedentary post-menopausal women (Earnest et al. 2008) and after a 6-month aerobic training program performed at moderate–vigorous intensity in sedentary healthy older and young men (Levy et al. 1998). In contrast, another study reported no effect on HRV parameters in response to a 2-week HIIT intervention and after a 2-week moderate-intensity aerobic training program in physically inactive adults (Alansare et al. 2018). These controversies could be explained by the different training programs’ duration (2 weeks vs 12 weeks) and also by the relatively small sample size (*n* = 13) of the study conducted by Alansare et al. (Alansare et al. 2018), which can be underpowered to identify changes in HRV between groups (Earnest et al. 2008). A further explanation for these discrepancies between studies could be the obvious differences in the exercise intervention designs since we included resistance training sessions in both PAR and HIIT groups once and twice a week, respectively, and this training modality could not affect HRV in sedentary middle-aged adults according to a review article (Kingsley and Figueroa 2016). Also, age groups (young adults vs middle-aged adults) and methodological considerations (Plaza-Florido et al. 2021) during the HRV assessment and processing could justify these differences.

Our study shows that a 12-week HIIT intervention increased SDNN (i.e., an indicator of global autonomic modulation) and RMSSD (i.e., an indicator of vagal modulation), decreased the SS (i.e., an indicator of sympathetic activity), and reduced, with a dependence of heart rate, the S/PS ratio (i.e., an indicator of autonomic balance) (Navarro-Lomas et al. 2020). Overall, these findings suggest a higher vagal tone during resting conditions after a 12-week exercise intervention based on HIIT. Similar results were reported after a 12-week (Ramírez-Vélez et al. 2020) and a 2-week (Alansare et al. 2018) training programs in physically inactive adults. Moreover, a 6-month HIIT program improves HRV parameters, including SDNN and RMSSD, in middle-aged adults after a coronary intervention (Munk et al. 2010). Interestingly, we identified similar effects of PAR and HIIT exercise interventions in HRV parameters.

Our results also show similar changes in HRV parameters after a 12-week HIIT + EMS program, which could be explained by an enhancement of the sympathovagal balance as a consequence of the HIIT + EMS program (Ricci et al. 2020). The effects of EMS training on HRV have been recently investigated in obese patients after bariatric surgery, observing no significant differences compared with a control group (Ricci et al. 2020). Our results show that an HIIT + EMS intervention did not promote additional improvements in HRV, apart from those obtained by an HIIT intervention alone, which concur with those obtained by the above-mentioned study (Ricci et al. 2020). Therefore, an HIIT + EMS training intervention seems to be a non-effective strategy to improve HRV (RMSSD, SDNN) in sedentary middle-aged adults.

Physical exercise is a well-recognized tool to improve several health-related outcomes (e.g., body composition (Amaro-Gahete et al. 2019b) or cardiometabolic risk (Amaro-Gahete et al. 2019a), among others). Understanding the influence of physical exercise on these components and its interaction with exercise-related changes in HRV is of clinical interest. We observed a significant association between changes in HRV parameters (i.e., increases in SDNN and RMSSD; and decreases in SS and S/PS ratio) and changes in body composition (i.e., FMI, VAT and LMI, but not BMI) after the intervention. Similar findings have been previously reported by Tian et al. (2015) who found an inverse association between HRV parameters and fat mass after a training program in adults with overweight/obesity, which could be explained by adiposity influences over autonomic function (Tian et al. 2015; Plaza-Florido et al. 2019). Also, improvements in lean mass have been related to increments in vagal tone (Andrew et al. 2013). The autocrine, paracrine, and endocrine actions of myokines over cardiometabolic health (i.e., lipolysis, insulin sensitivity or general metabolism) (Barbalho et al. 2020) could justify this association. Interestingly, we do not observe associations between exercise-induced changes in HRV parameters with exercise-induced changes in BMI. Although vagal-related HRV parameters have been negatively related to BMI (Koenig et al. 2014), similar BMI values could account for different fat and lean mass data (Felber Dietrich et al. 2008). Hence, to analyze changes in body composition after different training programs, FMI, VAT and LMI seem to be more appropriate parameters than BMI as direct indicators of total fat mass, visceral fat and lean mass, whose associations with cardiac autonomic function have been previously discussed. Furthermore, we found an association between exercise-related changes in HRV (i.e., increments in SDNN and reductions in SS and S/PS) and a decrease in cardiometabolic risk. To the best of our knowledge, this association has not been directly tested, but a recent narrative review suggests that increased vagal-related HRV parameters could be related to a decreased cardiometabolic risk after an exercise intervention (Thayer et al. 2010) which is in line with the present findings. Future studies are needed to well-understand this relationship.

The present study had some limitations. The present analyses have an exploratory nature and further studies are needed to investigate whether changes in HRV in response to different training modalities have the same magnitude or not. Moreover, our results should not be extrapolated to other populations since we only included sedentary middle-aged adults (i.e., 45–65 years old). In addition, although ≈ 75% of the women participants were post-menopausal, we did not assess ovarian hormones in the present study, a fact that could be a potential confounder (Martins et al. 2001; Bai et al. 2009). Finally, although the influence of different types of breathing over HRV is not clear (Wessel et al. 2009), some studies have found that paced vs. spontaneous breathing can differently affect HRV parameters (Hill et al. 2009; Plaza-Florido et al. 2021). To facilitate the resting status of the participants, we decided not to control their breathing. Further studies are needed to check whether the present findings apply to studies that control participants’ breathing during HRV assessment.

### Conclusion

Our study suggests that different exercise intervention modalities (i.e., PAR, HIIT and HIIT + EMS) induced an enhancement of HRV parameters (i.e., SDNN, RMSSD, SS and S/PS ratio) in sedentary middle-aged adults. Furthermore, we showed that exercise-related changes in HRV parameters (i.e., SDNN, RMSSD, SS and S/PS ratio) were associated with changes in body composition (i.e., LMI, FMI and VAT) and cardiometabolic risk after the intervention program.

## Supplementary Information

Below is the link to the electronic supplementary material.

Supplementary file1 (DOCX 615 KB)

## Abbreviations

ANOVA: Analysis of variance
ANCOVA: Analysis of covariance
*Β*: Standardized regression coefficient
BMI: Body mass index
CONSORT: Consolidated standards of reporting trials
EMS: Whole body electromyostimulation training
FMI: Fat mass index
HIIT: High-intensity interval training
HIIT + EMS: A concurrent training (aerobic + resistance training) based on physical activity recommendation from the World Health Organization
HRV: Heart rate variability
HDL-C: High-density lipoprotein cholesterol
Ln: Napierian logarithm
iMUDS: Sport and Health University Research Institute
LMI: Lean mass index
RMSSD: Square root of the mean squared differences between successive RR intervals
*R*^2^: Adjusted determination coefficient
SDNN: Standard deviation of RR intervals
SS: Stress score
S/PS ratio: Sympathetic/parasympathetic ratio
SD1: Standard deviation of Poincare plot orthogonal to the line-of-identity
SD2: Standard deviation of Poincare plot along the line-of-identity
VAT: Visceral adipose tissue volume
